# Onodera’s prognostic nutritional index is a strong prognostic indicator for patients with hepatocellular carcinoma after initial hepatectomy, especially patients with preserved liver function

**DOI:** 10.1186/s12893-020-00917-2

**Published:** 2020-10-31

**Authors:** Akihiro Tanemura, Shugo Mizuno, Aoi Hayasaki, Kazuyuki Gyoten, Takehiro Fujii, Yusuke Iizawa, Hiroyuki Kato, Yasuhiro Murata, Naohisa Kuriyama, Masashi Kishiwada, Hiroyuki Sakurai, Shuji Isaji

**Affiliations:** 1 Department of Hepatobiliary Pancreatic and Transplant Surgery, Mie University Graduate School of Medicine, 2-174 Edobashi, Tsu, Mie 514-0001 Japan; 2 Mie University Hospital, 2-174 Edobashi, Tsu, Mie 514-0001 Japan

**Keywords:** Hepatocellular carcinoma, PNI, Hepatectomy, Liver functional reserve, Technetium-99 m-diethylenetriaminepentaacetic acid-galactosyl-human serum albumin (99mTc-GSA) liver scintigraphy, LHL15, NLR, PLR, ALBI grade, GPS

## Abstract

**Background:**

Several inflammation-based scores are used to assess the surgical outcomes of hepatocellular carcinoma (HCC). The aim of the present study was to elucidate the prognostic value of the prognostic nutritional index (PNI) in HCC patients who underwent hepatectomy with special attention to preoperative liver functional reserve.

**Methods:**

Preoperative demographic and tumor-related factors were analyzed in 189 patients with HCC undergoing initial hepatectomy from August 2005 to May 2016 to identify significant prognostic factors.

**Results:**

Multivariate analysis for overall survival (OS) revealed that female sex (p = 0.005), tumor size (p < 0.001) and PNI (p = 0.001) were independent prognostic factors. Compared to the High PNI group (PNI ≥ 37, n = 172), the Low PNI group (PNI < 37, n = 17) had impaired liver function and significantly poorer OS (13% vs. 67% in 5-year OS, p = 0.001) and recurrence-free survival (RFS) (8 vs. 25 months in median PFS time, p = 0.002). In the subgroup of patients with a preserved liver function of LHL15 ≥ 0.9, PNI was also independent prognostic factor, and OS (21% vs. 70% in 5-year OS, p = 0.008) and RFS (8 vs. 28 months in median PFS time, p = 0.018) were significantly poorer in the Low PNI group than the High PNI group.

**Conclusions:**

PNI was an independent prognostic factor for HCC patients who underwent hepatectomy. Patients with PNI lower than 37 were at high risk for early recurrence and poor patient survival, especially in the patients with preserved liver function of LHL ≥ 0.9.

## Background

The carcinogenesis of hepatocellular carcinoma (HCC) is a multifactorial and multistep process that is associated with chronically inflamed liver parenchyma as a result of exposure to pro-inflammatory stimuli, such as hepatitis virus infection, ethanol consumption and steatohepatitis. Liver function in the patients with HCC also deteriorates with tumor progression. Therefore, compared to other malignancies, the prognosis of patients with HCC is highly influenced by tumor extension and the severity of the underlying liver function.

In addition to tumor biological and liver functional status, there is increasing evidence that several inflammation-based scores predict the prognosis of patients with malignancies, including HCC, because the host inflammatory response plays an important role in carcinogenesis and progression via the enhancement of proliferative signals, facilitation of angiogenesis, and promotion of invasion and metastasis. The indices or scores include the neutrophil-to-lymphocyte ratio (NLR), platelet-to-lymphocyte ratio (PLR), Glasgow Prognostic Score (GPS), and Onodera’s prognostic nutritional index (PNI), which are easily calculated from simple and low-cost blood tests [1–4]. PNI and GPS consist of albumin and lymphocytes or C-reactive protein (CRP), which may reflect the balance between the pro-tumor inflammatory status and nutritional status. Furthermore, the component of PNI and GPS, albumin, also reflects impaired protein synthesis secondary to chronic liver disease in HCC. Buzby et al. [5] originally reported PNI in 1980 to predict the development of postoperative complications after abdominal and thoracic surgery, and Onodera et al. [4] simplified this index in 1984. Since the 2010s, Onodera’s PNI has been widely used as a predictor of patient survival in various malignant tumors, including gastrointestinal [6–9] and nongastrointestinal cancers [10–12].

Several previous studies [13–21] revealed the prognostic significance of PNI in HCC patients, which consists of albumin and lymphocytes. However, none of these studies clearly examined why low PNI correlated to the prognosis. Low PNI means hypoalbuminemia and/or lymphocytopenia. Low albumin levels reflect malnutrition and an impaired ability of protein synthesis in the liver, i.e., impaired liver function. Lymphocytepenia contributes to tumor development and progression. Therefore, liver function strongly affects the prognostic significance of PNI in HCC patients. To the best of our knowledge, no studies evaluated the prognostic significance of PNI in HCC patients based on liver function.

The aim of present study was to clarify the prognostic values of PNI in HCC patients who underwent initial hepatectomy without previous treatment with special attention to preoperative liver function.

## Methods

### Subjects

We reviewed the database of primary HCC patients who underwent initial hepatectomy at Mie University Hospital and identified 212 consecutive cases from August 2005 to May 2016. The radiological diagnosis of HCC was made preoperatively based on the findings of dynamic enhanced CT and confirmed histologically in resected specimens, except for cases with total necrosis from preoperative trans-arterial chemoembolization (TACE). In this study, TACE was performed in 109 patients as preoperative treatment for initial hepatectomy, of whom 24 cases showed total necrosis. The 8 patients who were ultimately diagnosed with combined hepatocellular-cholangiocarcinoma (n = 6), undifferentiated carcinoma (n = 1) and carcinosarcoma (n = 1) were excluded from this study. Fifteen patients were also excluded from this study because of a lack of data on total lymphocyte count or liver functional assessment, such as indocyanine green retention retention test at 15 min (ICGR15) and GSA uptake ratio of the liver to the liver plus heart at 15 min (LHL15) in Technetium-99 m-diethylenetriaminepentaacetic acid-galactosyl-human serum albumin (99mTc-GSA) liver scintigraphy. Finally, 189 patients were included as subjects in this study (Additional file 1). The study protocol was approved by medical ethics committee of Mie University Hospital (No. 3173). The study protocol was announced on the hospital's homepage to provide the opportunity for patients to refuse participation.

Laboratory data obtained just before surgery was used to assess the preoperative demographic data, standard liver biochemistry, and tumor malignancy, including serum alpha fetoprotein (AFP) and des-γ-carboxyprothrombin (DCP). All patients received the ICG test and 99mTc-GSA liver scintigraphy to assess the liver functional reserve. PNI was calculated from serum albumin level and lymphocyte count as described in the previous literature: 10 × albumin (g/dl) + 0.005 × total lymphocyte count (per mm3) [4]. NLR was calculated as total neutrophil count (per mm3)/total lymphocyte count (per mm3) [1]. PLR was calculated as platelet count (per mm3)/total lymphocyte count (per mm3) [2]. The albumin-bilirubin (ALBI) grade was calculated according to the formula described in the original paper [22]. Surgical outcomes were assessed using operation time, blood loss, postoperative complications using the Clavien-Dindo classification, and postoperative laboratory data related to liver function (total bilirubin and prothrombin time international normalized ratio (PT-INR)) on postoperative day (POD) 5.

### Determination of the type of hepatectomy

After HCC diagnosis, the most appropriate surgical procedure was determined based on the tumor size, location, and the liver functional reserve as judged from the findings of ICGR15 and GSA uptake ratio of LHL15, as described in our previous reports [23, 24].

### Patient follow-up after hepatectomy

Follow-up after surgery included periodic blood tests and monitoring of tumor markers (AFP and DCP levels). Dynamic CT images and/or MRI of the remnant liver were performed every 3–4 months for 2 years after hepatectomy and every 6 months thereafter. Chest CT, whole abdominal CT, brain MRI, and bone scintigraphy were performed if recurrence of extrahepatic HCC was suspected.

### Statistical analyses

Categorical and continuous data were compared between groups using chi-squared and Mann–Whitney *U* tests, respectively. Continuous data are shown as medians and ranges. Patient survival was compared using Kaplan–Meier curves, and differences in survival between groups were analyzed using the log–rank test. In evaluating factors affecting OS, the Cox regression model with stepwise variable selection was used for multivariate analysis. The optimum PNI cut-off value was determined via comparisons of OS in Kaplan–Meier curves at each PNI cut-off value changing by one from 35 to 50. In addition, Evaluate Cutpoints [25] was used to evaluate the optimal cut-off value for OS. Differences were considered significant at p < 0.05. The day of final follow-up was November 31, 2016, and there was no loss to follow-up.

## Result

### Prognostic factor analysis in the whole patients

**Fig. 1:**
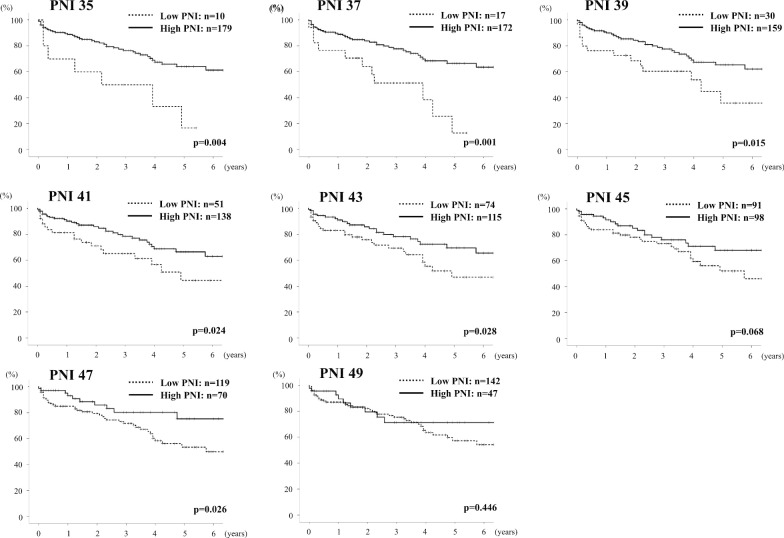
Overall survival (OS) at each PNI cut-off value of 35 to 50

The preoperative demographic and clinical characteristics are described in Table 1. Histological examination of the resected specimen and surgical outcomes are shown in Table 2. Preoperatively detectable clinical parameters were analyzed using univariate analysis to identify prognostic factors for overall survival (OS). Female sex (p = 0.023), albumin (p = 0.003), tumor size (p < 0.001), beyond Milan criteria (p = 0.002) and PNI (p = 0.02) were significantly associated with poor OS in univariate analysis. Multivariate analysis revealed that female sex (p = 0.005), tumor size (p < 0.001) and PNI (p = 0.001) were independent prognostic factors (Table 3). Therefore, we examined PNI as the most valuable index of the various inflammation-based prognostic scores. We compared surgical outcomes and survival by dividing patients into two groups according to PNI value. The optimum PNI cut-off value was determined via comparisons of OS in Kaplan–Meier curves at each PNI cut-off value of 35 to 50, and the most significant value that discriminated survival was determined as PNI 37 (Fig. 1). In addition, we evaluated PNI cut-off using application “Evaluate Cutpoints” described in the paper which evaluates optimal cutpoint of the continuous covariate in survival analysis [25]. As a result, PNI 37.25 was defined as an optimal cutpoint, that coincided with the cut-off value from manual method comparing the Kaplan–Meier curves.

**Table 1:** Preoperative demographics and clinical data (preoperative prognostic factors) in the 189 patients with HCC

Variables	
Age (years)	70 (41–85)
Sex (male/female)	153 (81%)/36 (19%)
BMI	22.1 (13.2–34.5)
Lymphocyte count (/mm^3^)	1400 (320–3950)
Neutrophil count (/mm^3^)	3020 (820–8780)
Platelet count (10^3^/µl)	18.3 (4.1–219.0)
Albumin (g/dl)	3.8 (2.3–5.3)
Total bilirubin (mg/dl)	0.6 (0.2–2.5)
PT-INR	1.05 (0.87–1.41)
Child–Pugh A/B/C	178 (94.2%) / 11 (5.8%) / 0
AFP (ng/ml)	12 (1–253,875)
DCP (mAU/ml)	115 (1–286,400)
ICG R15 (%)	12.9 (0.3–76.3)
LHL15	0.935 (0.679–0.987)
Tumor size (cm)	4.0 (0.5–24.0)
Multiple tumor	49 (25.9%)
BCLC stage 0/A/B/C	17 / 121 / 38 / 13
Milan criteria within / beyond	107 (56.6%) / 82 (43.4%)
PNI	45.4 (26.0–60.0)
NLR	2.06 (0.52–14.2)
PLR	135 (33–1685)
ALBI grade 1 / 2 / 3	85 (45%) / 99 (52%) / 5 (3%)
Underlying liver disease	
NBNC / HBV / HCV	85 (45%) / 26 (14%) / 78 (41%)

*BMI* body mass index, *PNI* prognostic nutritional index, *PT-INR* prothrombin time-international normalized ratio, *AFP* alpha fetoprotein, *DCP* des-γ-carboxyprothrombin, *ICGR15* indocyanine green retention rate at 15 min, *LHL15* GSA uptake ratio of the liver to the liver plus heart at 15 min, *BCLC stage* Barcelona Clinic Liver Cancer stage, *NLR* neutrophil-to-lymphocyte ratio, *PLR* platelet-to-lymphocyte ratio, *ALBI grade* albumin-bilirubin grade, *NBNC* non-B non-C, *HBV* hepatits B, *HCV* hepatitis C

**Table 2:** Histological examination of the resected specimen and surgical outcomes in the 189 patients with HCC

Variables	
*Tumor differentiation*	
Well	42 (22%)
Moderately	90 (48%)
Poorly	26 (14%)
*Vascular invasion*	
vp1/vp2/vp3/vp4	51 / 3 / 7 / 3
vv ( +)	12 (6.3%)
*Liver histology*	
Normal liver	24 (13%)
Chronic hepatitis	77 (41%)
Liver cirrhosis	70 (37%)
Unknown	18 (9%)
Operation time (minutes)	336 (127–983)
Blood loss (ml)	1120 (0–36,000)
2 or more sectionectomy	57 (30%)
PT-INR on POD5	1.13 (0.92–1.71)
Total bilirubin on POD5 (mg/dl)	0.9 (0.3–11.2)
Complications (C-D ≥ IIIa)	38 (20%)
Inhospital mortality	10 (5.3%)

*PT-INR* prothrombin time-international normalized ratio, POD: postoperative day, *C–D* Clavien–Dindo, *vp1* microvascular portal vein invasion, *vp2* portal vein tumor thrombus in distal to the second order branches of portal vein, *vp3* portal vein tumor thrombus in the first branch of portal vein, *vp4* portal vein tumor thrombus in the main portal trunk or the opposite-side portal branch, *vv ( +)* microvascular hepatic vein invasion

**Table 3:** Uni- and multivariate analysis to identify preoperative prognostic factors associated with overall survival in the 189 patients with HCC

Variables	Univariate analysis		Multivariate analysis	
	HR (95% CI)	p value	HR (95% CI)	p value
Age	1.014 (0.98–1.048)	0.41		
Female	*0.3 (0.109–0.847)*	*0.023*	*0.22 (0.076–0.62)*	*0.005*
BMI	0.97 (0.89–1.06)	0.50		
Lymphocyte count (/mm^3^)	1.0 (1.00–1.00)	0.26		
Neutrophil count (/mm^3^)	1.0 (1.00–1.00)	0.61		
Platelet count (10^3^/µl)	1.0 (0.98–1.02)	0.97		
Albumin (g/dl)	*0.44 (0.26–0.76)*	*0.003*		
Total bilirubin (mg/dl)	0.26 (0.06–1.02)	0.054		
PT-INR	4.30 (0.05–61.06)	0.78		
Child–Pugh B	1.89 (0.45–7.93)	0.38		
AFP (ng/ml)	1.0 (1.00–1.00)	0.72		
DCP (mAU/ml)	1.0 (1.00–1.00)	0.89		
ICG R15 (%)	1.03 (1.00–1.059)	0.058		
LHL15	2.57 (0.00–32,477,422)	0.91		
Tumor size (cm)	*1.14 (1.08–1.21)*	* < 0.001*	*1.11 (1.05–1.18)*	* < 0.001*
Multiple tumor	1.03 (0.37–2.29)	0.94		
Milan criteria beyond	*3.25 (1.51–6.99)*	*0.002*		
PNI	*0.93 (0.88–0.99)*	*0.02*	*0.92 (0.88–0.97)*	*0.001*
NLR	1.09 (0.96–1.25)	0.19		
PLR	1.0 (1.00–1.00)	0.55		
ALBI score	2.22 (0.99–5.08)	0.06		

Differences were considered significant at p < 0.05, shown as italic values

*BMI* body mass index, *PNI* prognostic nutritional index, *PT-INR* prothrombin time-international normalized ratio, *AFP* alpha fetoprotein, *DCP* des-γ-carboxyprothrombin, *ICGR15* indocyanine green retention rate at 15 min, *LHL15* GSA uptake ratio of the liver to the liver plus heart at 15 min, *NLR* neutrophil-to-lymphocyte ratio, *PLR* platelet-to-lymphocyte ratio, *ALBI score* albumin-bilirubin score

### Comparison of patient characteristics and outcomes between Low and High PNI groups in all patients

**Fig. 2:**
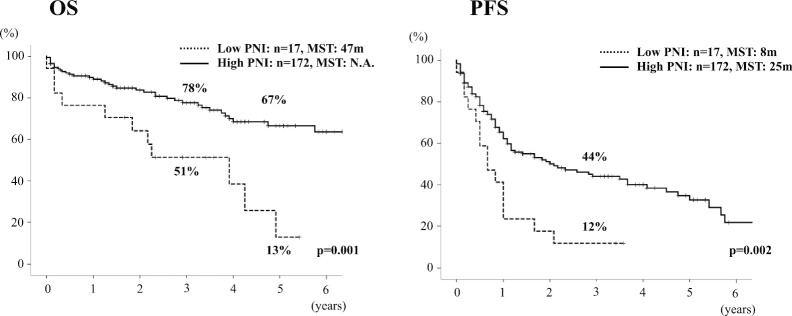
Overall (OS) and progression free survivals (PFS) according to PNI cut-off value of 37

The 189 patients were divided into two groups based on PNI score: Low PNI group (n = 17): PNI < 37 and High PNI group (n = 172): PNI ≥ 37. Patient background and surgical outcomes were compared between the two groups. Albumin level and total lymphocyte count were significantly lower in the Low PNI group than the High PNI group (p < 0.001) because both factors are main component of PNI. For the assessment of liver function, PT-INR, ICGR15, the rate of Child–Pugh B and ALBI grade ≥ 2 were significantly higher, and LHL15 was significantly lower in the Low PNI group than the High PNI group (p < 0.001, p = 0.007, p < 0.001, p < 0.001 and p < 0.001, respectively), which indicates that patients in the Low PNI group had impaired liver function. NLR was significantly higher in the Low PNI group than the High PNI group (p = 0.004), which indicates that patients in the Low PNI group were an immunocompromised host (Table 4). For tumor biology, AFP was significantly higher in the Low PNI group than the High PNI group (p = 0.05). However, the other factors were not different between the two groups, such as tumor morphology, Barcelona Clinic Liver Cancer (BCLC) stage, and histological findings. For intra- and postoperative outcomes, PT-INR and total bilirubin level on POD 5 were significantly higher in the Low PNI group than in the High PNI group (p = 0.03 and p = 0.012, respectively), which indicates that liver functional recovery was delayed in patients with Low PNI (Table 5). OS was significantly lower in the Low PNI group than the High PNI group and showed 5-year OS of 13% and 67%, respectively (p = 0.001). Progression-free survival (PFS) was also significantly lower in the Low PNI group than the High PNI group and showed median PFS time of 8 and 25 months, respectively (p = 0.002) (Fig. 2). The median PFS time in the Low-PNI group showed quite a poor outcome of less than 1 year.

**Table 4:** Comparison of patient characteristics between Low and High PNI groups

PNI median	Low PNI (PNI < 37: n = 17) 33.6 (26.0–36.9)	High PNI (PNI ≥ 37: n = 172) 45.9 (37.3–60.0)	p value
Age	68 (47–81)	70 (41–85)	n.s
Sex (male / female)	11/6	142/30	n.s
BMI	23.2 (15.6–29.6)	22.1 (13.2–32.5)	n.s
Lymphocyte count (/mm^**3**^**)**	*710 (320–2260)*	*1480 (350–2950)*	* < 0.001*
Neutrophil count (/mm^3^)	2420 (910–8780)	3030 (820–8680)	n.s
Albumin (g/dl)	*2.9 (2.3–3.4)*	*3.8 (2.7–5.3)*	* < 0.001*
Total bilirubin (mg/dl)	0.6 (0.4–1.6)	0.6 (0.2–2.5)	n.s
Platelet count (10^3^/µl)	13.4 (4.4–37.9)	18.6 (4.1–219.0)	n.s
PT-INR	*1.16 (0.87–1.41)*	*1.05 (0.88–1.34)*	* < 0.001*
Child–Pugh A/B/C	*11 / 6 / 0*	*167 / 5 / 0*	* < 0.001*
ICG R15 (%)	*23.9 (6.3–49.9)*	*12.6 (0.3–76.3)*	*0.007*
LHL15	*0.87 (0.68–0.96)*	*0.94 (0.76–0.99)*	* < 0.001*
ALBI grade 1 / 2 / 3	*0 / 12 / 5*	*85 / 87 / 0*	* < 0.001*
NLR	*3.98 (0.86–14.16)*	*2.00 (0.52–12.8)*	*0.004*
PLR	223 (49–486)	134 (23–1685)	n.s
Underlying liver disease			*0.03*
Hepatitis B	2 (12%)	24 (14%)	
Hepatitis C	12 (70%)	66 (38%)	
Non-B non-C	3 (18%)	82 (48%)	
Liver pathology			n.s
Normal liver	1 (6%)	23 (15%)	
Chronic hepatitis	5 (29%)	72 (47%)	
Liver cirrhosis	11 (65%)	59 (38%)	

Differences were considered significant at p < 0.05, shown as italic values

*BMI* body mass index, *PNI* prognostic nutritional index, *ICGR15* indocyanine green retention rate at 15 min, *LHL15* GSA uptake ratio of the liver to the liver plus heart at 15 min, *ALBI score* albumin-bilirubin score, *NLR* neutrophil-to-lymphocyte ratio, *PLR* platelet-to-lymphocyte ratio

**Table 5:** Tumor characteristics and surgical outcomes in Low and High PNI groups

	Low PNI (PNI < 37: n = 17)	High PNI (PNI ≥ 37: n = 172)	p value
PNI median	33.6 (26.0–36.9)	45.9 (37.3–60.0)	
Tumor size (cm)	4.7 (1.1–15.0)	4.0 (0.5–24.0)	n.s
Multiple tumor	7 (41%)	42 (24%)	n.s
AFP (ng/ml)	*62 (2–60,105)*	*11 (1–253,875)*	*0.05*
DCP (mAU/ml)	1094 (14–85,330)	92 (1–286,400)	n.s
BCLC stage			n.s
0	0 (0%)	17 (10%)	
A	13 (76%)	108 (63%)	
B	3 (18%)	35 (20%)	
C	1 (6%)	12 (7%)	
Within Milan criteria	9 (53%)	98 (57%)	n.s
Tumor differentiation			
Well	2 (13%)	40 (28%)	n.s
Moderately	10 (62%)	80 (56%)	
Poorly	4 (25%)	22 (16%)	
Vascular invasion			
vp ( +)	9 (56%)	55 (38%)	n.s
vv ( +)	1 (6%)	11 (8%)	n.s
Operation time (minutes)	336 (159–526)	335 (127–983)	n.s
Blood loss (ml)	1711 (520–10,382)	1080 (0–36,000)	n.s
≥ 2 sectionectomy	3 (18%)	54 (31%)	n.s
Complications (C-D ≥ IIIa)	5 (29%)	33 (20%)	n.s
Mortality	2 (12%)	8 (5%)	n.s
PT-INR on POD5	*1.20 (0.98–1.71)*	*1.12 (0.92–1.52)*	*0.03*
Total bilirubin on POD5 (mg/dl)	*1.5 (0.5–11.2)*	*0.9 (0.3–8.3)*	*0.012*

Differences were considered significant at p < 0.05, shown as italic values

*AFP* alpha fetoprotein, *DCP* des-γ-carboxyprothrombin, *BCLC stage* Barcelona clinic liver cancer stage, *C–D* Clavien–Dindo, *POD* postoperative day, *PT-INR* prothrombin time-international normalized ratio, *vp ( +)* portal vein invasion including microvascular invasion, *vv ( +)* hepatic vein invasion including microvascular invasion

### Surgical outcomes according to LHL15

**Fig. 3:**
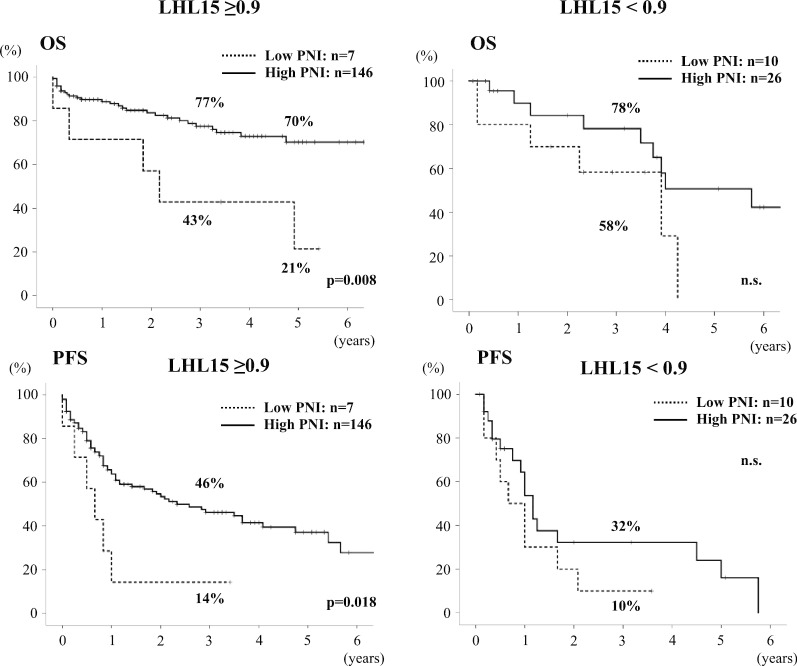
Overall (OS) and progression free survivals (PFS) according to PNI cut-off value of 37 in subgroup according to liver functional reserve using LHL15

PNI correlated to preoperative liver function because the main component of PNI, albumin level, also reflects the protein synthesis ability of the liver. Therefore, we examined the subgroup with relatively preserved liver function. We adopted 99mTc-GSA liver scintigraphy as a better predictor of liver functional reserve rather than ICG test, because in previous studies, LHL15 had significant correlation with hyaluronic acid, platelet count and histological findings of liver, while ICGR15 does not always represent accurate hepatic functional reserve [23, 26]. In the subgroup with LHL15 ≥ 0.9 (n = 153), which cut-off was defined according to our previous study [23], female sex (p = 0.04), albumin (p = 0.017), ICGR15 (p = 0.008), tumor size (p < 0.001), beyond Milan criteria (p = 0.006), PNI (p = 0.003) and ALBI score (p = 0.01) were significantly associated with poor OS in univariate analysis. Multivariate analysis revealed that PNI remained an independent prognostic factor (p = 0.005) with female sex (p = 0.011) and tumor size (p < 0.001) (Table 6). In the subgroup with LHL15 < 0.9 (n = 36), DCP (p = 0.005) and multiple tumor (p = 0.035), but not PNI, were independent prognostic factors in multivariate analysis (Table 7). In comparisons of the Low- and High PNI groups using the cut-off value of 37 in the subgroup with LHL15 ≥ 0.9, similar to the analysis of all patients, the Low PNI group had significantly higher PT-INR, lower PLR, a higher rate of ALBI grade 1 and higher total bilirubin level on POD5 than the High PNI group (p = 0.018, p = 0.022, p = 0.001 and p = 0.024, respectively) (Tables 8 and 9). OS and PFS were significantly lower in the Low PNI group than the High PNI group and showed 5-year OS of 21% vs. 70% in OS (p = 0.008) and median PFS time of 8 and 28 months (p = 0.018) in the subgroup with LHL15 ≥ 0.9, respectively, but these factors were not different in the subgroup with LHL < 0.9 (Fig. 3).

**Table 6:** Uni- and Multivariate analysis to identify preoperative prognostic factors associated with overall survival in the 153 patients with LHL15 ≥ 0.9

Variables	Univariate analysis		Multivariate analysis	
	HR (95% CI)	p value	HR (95% CI)	p value
Age	1.018 (0.98–1.06)	0.40		
Female	*0.4 (0.05–0.93)*	*0.04*	*0.15 (0.034–0.65)*	*0.011*
BMI	1.0 (0.90–1.12)	0.39		
Lymphocyte count (/mm^3^)	1.0 (1.00–1.00)	0.26		
Neutrophil count (/mm^3^)	1.0 (1.00–1.00)	0.37		
Platelet count (10^3^/µl)	1.0 (0.99–1.03)	0.63		
Albumin (g/dl)	*0.42 (0.20–0.86)*	*0.017*		
Total bilirubin (mg/dl)	0.76 (0.33–1.79)	0.054		
PT-INR	4.30 (0.35–53.19)	0.26		
Child–Pugh B	1.87 (0.74–4.72)	0.18		
AFP (ng/ml)	1.0 (1.00–1.00)	0.80		
DCP (mAU/ml)	1.0 (1.00–1.00)	0.28		
ICG R15 (%)	*1.03 (1.01–1.05)*	*0.008*		
LHL15	0.52 (0.00–6.69)	0.23		
Tumor size (cm)	*1.13 (1.07–1.18)*	* < 0.001*	*1.13 (1.06–1.21)*	* < 0.001*
Multiple tumor	1.30 (0.69–2.43)	0.41		
Milan criteria beyond	*2.25 (1.26–4.00)*	*0.006*		
PNI	*0.94 (0.90–0.98)*	*0.003*	*0.92 (0.87–0.97)*	*0.005*
NLR	1.08 (0.98–1.20)	0.12		
PLR	1.0 (1.00–1.00)	0.58		
ALBI score	*2.08 (1.19–3.63)*	*0.01*		

Differences were considered significant at p < 0.05, shown as italic values

*BMI* body mass index, *PNI* prognostic nutritional index, *PT-INR* prothrombin time-international normalized ratio, *AFP* alpha fetoprotein, *DCP* des-γ-carboxyprothrombin, *ICGR15* indocyanine green retention rate at 15 min, *LHL15* GSA uptake ratio of the liver to the liver plus heart at 15 min, *NLR*: neutrophil-to-lymphocyte ratio, *PLR* platelet-to-lymphocyte ratio, *ALBI score* albumin-bilirubin score

**Table 7:** Uni- and multivariate analysis to identify preoperative prognostic factors associated with overall survival in the 36 patients with LHL15 < 0.9

Variables	Univariate analysis		Multivariate analysis	
	HR (95% CI)	p value	HR (95% CI)	p value
Age	1.005 (0.95–1.07)	0.87		
Female	0.59 (0.38–7.52)	0.49		
BMI	0.93 (0.82–1.06)	0.29		
Lymphocyte count (/mm^3^)	1.0 (1.00–1.00)	0.69		
Neutrophil count (/mm^3^)	1.0 (1.00–1.00)	0.73		
Platelet count (10^3^/µl)	1.0 (0.97–1.03)	0.90		
Albumin (g/dl)	0.52 (0.21–1.28)	0.16		
Total bilirubin (mg/dl)	1.12 (0.46–2.70)	0.81		
PT-INR	9.01 (0.18–461.3)	0.27		
Child–Pugh B	1.86 (0.49–7.04)	0.36		
AFP (ng/ml)	1.0 (1.00–1.00)	0.33		
DCP (mAU/ml)	*1.0 (1.00–1.00)*	*0.01*	*1.0 (1.00–1.00)*	*0.005*
ICG R15 (%)	1.03 (0.98–1.07)	0.22		
LHL15	7.68 (0.00–135,931)	0.68		
Tumor size (cm)	1.11 (0.98–1.26)	0.12		
Multiple tumor	2.82 (0.97–8.20)	0.058	*3.27 (1.09–9.90)*	*0.035*
Milan criteria beyond	1.52 (0.52–4.46)	0.45		
PNI	0.95 (0.88–1.02)	0.15		
NLR	1.05 (0.93–1.20)	0.44		
PLR	1.0 (1.00–1.00)	0.59		
ALBI score	1.72 (0.72–4.13)	0.23		

Differences were considered significant at p < 0.05, shown as italic values

*BMI* body mass index, *PNI* prognostic nutritional index, *PT-INR* prothrombin time-international normalized ratio, *AFP* alpha fetoprotein, *DCP* des-γ-carboxyprothrombin, *ICGR15* indocyanine green retention rate at 15 min, *LHL15* GSA uptake ratio of the liver to the liver plus heart at 15 min, *NLR* neutrophil-to-lymphocyte ratio, *PLR* platelet-to-lymphocyte ratio, *ALBI score* albumin-bilirubin score

**Table 8:** Patient characteristics s in Low and High PNI groups according to liver fictional preserve

	LHL15 ≥ 0.9			LHL15 < 0.9		
Variables	Low PNI (n = 7)	High PNI (n = 146)	p value	Low PNI (n = 10)	High PNI (n = 26)	p value
Age (years)	72 (57–77)	70 (41–85)	n.s	65 (47–81)	68 (47–83)	n.s
Sex (male / female)	*3 / 4*	*122 / 24*	*0.02*	8/2	20/6	n.s
BMI	21.7 (16.8–26.3)	22.1 (14.0–32.5)	n.s	24.6 (15.6–29.6)	22.1 (13.2–31.5)	n.s
Lymphocyte count (/mm^3^)	*710 (530–1290)*	*1455 (350–2930)*	*0.001*	*800 (320–2260)*	*1500 (650–2950)*	*0.002*
Neutrophil count (/mm^3^)	3190 (910–8780)	3160 (1020–8680)	n.s	2420 (1140–7170)	2225 (820–3610)	n.s
Platelet count (10^3^/µl)	23.6 (6.6–37.9)	18.9 (4.8–219.0)	n.s	12.2 (4.4–28.9)	14.1 (4.1–156.0)	n.s
Albumin (g/dl)	*3.1 (2.3–3.3)*	*3.9 (2.7–5.3)*	* < 0.001*	*2.8 (2.3–3.4)*	*3.7 (3.0–4.7)*	* < 0.001*
Total bilirubin (mg/dl)	0.5 (0.4–1.5)	0.6 (0.2–1.5)	n.s	0.85 (0.4–1.6)	0.6 (0.3–2.5)	n.s
PT-INR	*1.16 (0.87–1.30)*	*1.04 (0.88–1.28)*	*0.018*	1.16 (1.0–1.41)	1.10 (0.93–1.34)	n.s
Child–Pugh A/B/C	6 / 1 / 0	143 / 3 / 0	n.s	5 / 5 / 0	2 / 24 / 0	n.s
AFP (ng/ml)	40 (2–33,430)	9 (1–253,875)	n.s	122 (9–60,105)	29 (4–88,940)	n.s
DCP (mAU/ml)	1501 (28–85,330)	88 (1–286,400)	n.s	260 (14–29,182)	237 (10–174,400)	n.s
ICG R15 (%)	13.5 (6.3–28.8)	11.8 (0.3–76.3)	n.s	31.7 (9.9–49.9)	18.3 (7.0–42.0)	n.s
LHL15	0.935 (0.904–0.963)	0.940 (0.90–0.987)	n.s	*0.853 (0.679–0.885)*	*0.876 (0.758–0.898)*	*0.04*
Tumor size (cm)	7.0 (2.0–13.0)	4.0 (0.5–24.0)	n.s	3.0 (1.1–15.0)	3.5 (0.7–20.0)	n.s
Multiple tumor	2 (29%)	38 (26%)	n.s	5 (50%)	4 (15.4%)	n.s
BCLC stage 0/A/B/C	0 / 6 / 0 / 1	13 / 91 / 31 / 11	n.s	0 / 7 / 3 / 0	4 / 17 / 4 / 1	n.s
Within Milan criteria	2 (29%)	78 (53%)	n.s	7 (70%)	20 (76.9%)	n.s
PNI	*34.7(26.6–36.9)*	*46.2 (37.4–60.0)*	* < 0.001*	*33.6 (26.0–36.5)*	*44.3 (37.3–54.8)*	* < 0.001*
NLR	4.49 (0.85–8.58)	2.10 (0.52–12.8)	n.s	*3.20 (1.07–14.16)*	*1.54 (0.56–3.14)*	*0.001*
PLR	*231 (110–486)*	*138 (33–1685)*	*0.022*	189 (49–350)	91 (35–1033)	n.s
ALBI grade 1 / 2 / 3	*0 / 6 / 1*	*74 / 72 / 0*	*0.001*	*0 / 6 / 4*	*11 / 15 / 0*	*0.001*
Underlying liver disease						
NBNC / HBV / HCV	1 / 2 / 4	76 / 21 / 49	n.s	2 / 0 / 8	6 / 3 / 17	n.s

Differences were considered significant at p < 0.05, shown as italic values

*BMI* body mass index, *PNI* prognostic nutritional index, *PT-INR* prothrombin time-international normalized ratio, *AFP* alpha fetoprotein, *DCP* des-γ-carboxyprothrombin, *ICGR15* indocyanine green retention rate at 15 min, *LHL15* GSA uptake ratio of the liver to the liver plus heart at 15 min, *NLR* neutrophil-to-lymphocyte ratio, *PLR* platelet-to-lymphocyte ratio, *ALBI grade* Albumin-Bilirubin grade, *NBNC* Non-B non-C, *HBV* hepatits B, *HCV* hepatitis C

**Table 9:** Tumor characteristics and surgical outcomes in Low and High PNI groups according to liver fictional preserve

	LHL15 ≥ 0.9			LHL15 < 0.9		
Variables	Low PNI (n = 7)	High PNI (n = 146)	p value	Low PNI (n = 10)	High PNI (n = 26)	p value
Tumor size (cm)	7.0 (2.0–13.0)	4.0 (0.5–24.0)	n.s	3 (1.1–15.0)	3.5 (0.7–20.0)	n.s
Multiple tumor	2 (29%)	38 (26%)	n.s	5 (50%)	4 (15%)	n.s
AFP (ng/ml)	40 (2–33,430)	9 (1–253,875)	n.s	122 (9–60,105)	29 (4–88,940)	n.s
DCP (mAU/ml)	1501 (28–85,330)	88 (1–286,400)	n.s	260 (14–29,182)	237 (10–174,400)	n.s
BCLC stage			n.s			n.s
0	0 (0%)	13 (9%)		0 (0%)	4 (15%)	
A	6 (86%)	91 (62%)		7 (70%)	17 (65%)	
B	0 (0%)	31 (21%)		3 (30%)	4 (15%)	
C	1 (14%)	11 (8%)		0 (0%)	1 (4%)	
Within Milan criteria	9 (53%)	98 (57%)	n.s	7 (70%)	20 (77%)	n.s
Tumor differentiation			n.s			
Well	2 (29%)	34 (29%)		0 (0%)	6 (29%)	n.s
Moderately	3 (43%)	68 (56%)		7 (78%)	12 (57%)	
Poorly	2 (29%)	19 (16%)		2 (22%)	3 (14%)	
Vascular invasion						
vp ( +)	4 (57%)	48 (38%)	n.s	5 (56%)	7 (33%)	n.s
vv ( +)	1 (14%)	11 (9%)	n.s	0 (0%)	0 (0%)	n.s
Operation time (minutes)	361 (170–526)	350 (137–983)	n.s	277 (159–413)	274 (127–580)	n.s
Blood loss (ml)	2865 (520–10,382)	1064 (0–36,000)	n.s	1711 (1270–4192)	1500 (56–7341)	n.s
≥ 2 sectionectomy	3 (43%)	52 (36%)	n.s	0 (0%)	2 (8%)	n.s
Complications (C–D ≥ IIIa)	1 (14%)	29 (20%)	n.s	4 (40%)	4 (15%)	n.s
Mortality	1 (14%)	8 (6%)	n.s	1 (10%)	0 (0%)	n.s
PT-INR on POD5	1.19 (1.10–1.71)	1.12 (0.92–1.52)	n.s	1.21 (0.98–1.49)	1.18 (0.94–1.51)	n.s
Total bilirubin on POD5 (mg/dl)	*1.5 (0.7–11.2)*	*0.9 (0.3–8.3)*	*0.024*	1.2 (0.5–2.8)	1.0 (0.4–4.7)	n.s

Differences were considered significant at p < 0.05, shown as italic values

*AFP* alpha fetoprotein, *DCP* des-γ-carboxyprothrombin, *BCLC stage* Barcelona clinic liver cancer stage, *C–D* Clavien–Dindo, *POD* postoperative day, *vp ( +)* portal vein invasion including microvascular invasion, *vv ( +)* hepatic vein invasion including microvascular invasion

## Discussion

The present study determined that female sex, tumor size and PNI were independent prognostic factors for patients with hepatectomy for HCC without previous treatment. Other inflammation-based scores, such as NLR and PLR, were not independent prognostic factors. The optimum PNI cut-off value for OS was 37. Patients with a low PNI less than 37 had significantly impaired liver function, significantly poor prognosis and were immunocompromised. Even in patients who had preserved liver function with LHL ≥ 0.9, PNI remained an independent prognostic factor and showed significantly lower OS and PFS, which were not observed in patients with impaired liver function with LHL15 < 0.9.

Although several previous studies indicated that PNI was a significant prognostic factor in HCC patients [13–21], these reports did not examine why low PNI correlated to the prognosis. Because PNI consists of albumin and lymphocyte levels, low PNI means hypoalbuminemia and lymphocytopenia, which may contribute to tumor development and progression. Lower albumin levels in patients with lower PNI reflects malnutrition and impaired protein synthesis ability in livers with chronic inflammation and fibrosis due to the underlying liver disease. Serum albumin level was integrated into several HCC staging systems, and a new albumin-derived score, ALBI score [22], predicted the prognosis of HCC patients. In the present study, the Low PNI group had lower survival and poorer liver functions and showed significantly higher PT-INR, ICGR15, a higher rate of Child–Pugh B and ALBI grade ≥ 2 and significantly lower LHL15. Chan et al. [14] revealed that a lower PNI (< 45) group was associated with higher model for end-stage liver disease (MELD) score and a higher rate of Child–Pugh class B patients in early stage HCC patients. However, no previous studies examined the relationship between PNI and liver functional reserve tests, such as ICGR15 and LHL15. The ICG test and 99mTc-GSA liver scintigraphy are more beneficial to assess liver function in hepatectomy rather than the MELD score or Child–Pugh classification because these tests provide detailed liver functional data [23, 27]. Lower PNI was significantly associated with a detailed liver function in our study and showed significantly higher ICGR15 and lower LHL15.

Lymphocytes are another component and immune factor of PNI, and these cells play an important role in HCC recurrence and progression. We found a relationship between PNI and AFP, but not the other tumor biological statuses as assessed using tumor stage or histological findings. In a meta-analysis to systematically review the association between PNI and HCC prognosis [21], PNI was significantly associated with AFP level, tumor size and TNM stages. The loss of CD4 ( +) T lymphocytes strongly contributed to HCC development in a mouse model [28]. A clinical study on resected specimens of HCC patients demonstrated that high densities of CD3 ( +) and CD8 ( +) T cells in the interior and margins of the tumor were significantly associated with a low rate of recurrence and a prolonged RFS [29]. These findings indicate that host immune status with lymphocyte infiltration of the tumor is important to prevent tumor progression. The present study also examined lymphocyte-related markers, such as NLR and PLR, but these markers were not selected as an independent prognostic factors in multivariate analysis. PNI predicted the prognosis of HCC patient more precisely than NLR and PLR because the PNI contains albumin and lymphocyte levels as nutritional and immune factors.

Our study stratified the patient survival more clearly according to PNI compared to previous studies with the use of 37 as the cut off value. We determined 37 as the PNI cut-off value by comparing the survival curves stratified by various cut-off values from 35 to 50, and other studies of hepatectomized HCC used cut-off values that ranged from 45 to 53 [13–21]. One previous report defined the low-PNI group as PNI < 45, which had a significant adverse 5-year OS of 57% (n = 84) vs. 82% (n = 240), p = 0.001 [14]. Another study defined the low-PNI group as PNI < 48.5, which had a significant adverse 5-year OS of 46.5% (n = 122) vs. 76.5% (n = 134), p = 0.001 [15]. In contrast, our study newly defined the Low PNI group as PNI < 37, which had a more significant adverse 5-year OS of only 13% (n = 17) vs. 67% (n = 172), p = 0.001. When we compared PFS between the two groups, median PFS time in the Low PNI group was significantly shorter (8 months) than the High PNI group (25 months) (p = 0.002). Therefore, we determined that a PNI of 37 was the most practical cut-off value in clinical settings. When encountering HCC patients with PNI < 37, we should strictly consider their operative indication because they have a high risk of early recurrence after hepatectomy and poor prognosis. Preoperative immuno-nutritional management should be required to increase PNI when surgical resection is the sole curative treatment option.

It is apparent that PNI is influenced by the liver functional reserve because a serum level of albumin is one component of PNI, and albumin is synthesized in the liver. Thus, we considered that in the subgroup of patients with preserved liver function, which means that the ability of synthesis of albumin is relatively preserved, PNI would more accurately reflect pro-tumor inflammatory and nutritional status than in those with impaired liver function. Therefore, we performed a subgroup analysis that adjusted for liver functional reserve. The patients were divided into subgroups according to liver functional reserve using LHL15. Multivariate analysis revealed that PNI still remained an independent prognostic factor in the subgroup with LHL15 ≥ 0.9 but not in the subgroup with LHL15 < 0.9. In the preserved liver functional groups of LHL15 ≥ 0.9, the Low PNI group showed significantly lower OS and PFS than the High PNI group, but PNI showed no survival difference in the poor liver functional group of LHL < 0.9. These findings demonstrated that PNI was a useful practical tool, especially in patients with preserved liver functional reserve, but not in the patients with poor liver function. PNI was proven to be a strong prognostic factor especially in the patients who had liver function preserved well, because it worked well even in the patient subgroup with preserved liver function of LHL15 ≥ 0.9. In the patients with poor liver functional reserve with LHL < 0.9, tumor malignancy, such as high DCP level, and multiple tumors significantly affected patient survival more than PNI based on the multivariate analysis.

The results of our study showed that PNI may be used as a predictor of patient prognosis and an indicator for preoperative nutritional management. Preoperative nutritional treatment may be important for patients with PNI lower than 37 to increase albumin level and lymphocyte count because prognosis was extremely poor in patients with preserved liver functional reserve. Sarcopenia was recently correlated with postoperative complications and survival in HCC patients [30–32]. To improve nutritional state and sarcopenia, preoperative nutritional intervention may be important. Nutritional intervention with branched-chain amino acid (BCAA)-enriched nutrient increased albumin levels and BCAA-to-tyrosine ratios before TACE for HCC patients [33]. For the surgical treatment for HCC, perioperative enteral nutrition improved the recovery of gastrointestinal function, reduced morbidity and shortened the length of postoperative hospital stay [34, 35]. In our study, postoperative complication rate was similar between the Low- and High PNI groups. However, the rate of major hepatectomy was higher in High PNI group than in Low PNI group. If surgical procedure is same in the two groups, poor nutritional status in Low PNI group may give the negative impact on postoperative course. The outcomes of preoperative management to improve PNI should be evaluated prospectively, including how preoperative nutritional support may improve PNI and surgical outcomes, including patient prognosis.

## Conclusions

In conclusion, PNI was an independent prognostic factor for HCC patients who underwent hepatectomy, especially patients with preserved liver functional reserve as assessed by 99mTc-GSA liver scintigraphy. Patients with PNI lower than 37 were at high risk of early recurrence and poor survival, but they were a minor population of the patients receiving hepatectomy for HCC. Therefore, we should follow these patients carefully after surgery. On the other hand, patients with PNI 37 or greater had a better prognosis after hepatectomy for HCC.

## Supplementary information

**Additional file 1.** Detailed data of HCC patients who underwent hepatectomy.

## Data Availability

The datasets used and/or analyzed during the current study are available from the corresponding author on reasonable request.

## Abbreviations

AFP: Alpha fetoprotein
ALBI grade: Albumin-bilirubin grade
BCAA: Branched-chain amino acid
BCLC stage: Barcelona clinic liver cancer stage
CRP: C-reactive protein
DCP: Des-γ-carboxyprothrombin
GPS: Glasgow prognostic score
HCC: Hepatocellular carcinoma
ICGR15: Indocyanine green retention retention test at 15 min
LHL15: GSA uptake ratio of the liver to the liver plus heart at 15 min
MELD score: Model for end-stage liver disease score
MRI: Magnetic resonance imaging
MST: Median survival times
NLR: Neutrophil-to-lymphocyte ratio
OS: Overall survival
PFS: Progression-free survival
PLR: Platelet-to-lymphocyte ratio
PNI: Prognostic nutritional index
POD: Postoperative day
PT-INR: Prothrombin time international normalized ratio
TACE: Trans-arterial chemoembolization
99mTc-GSA: Technetium-99 m-diethylenetriaminepentaacetic acid-galactosyl-human serum albumin

## References

[1] Walsh SR, Cook EJ, Goulder F, Justin TA, Keeling NJ (2005). Neutrophil-lymphocyte ratio as a prognostic factor in colorectal cancer. J Surg Oncol.

[2] Smith RA, Bosonnet L, Ghaneh P, Sutton R, Evans J, Healey P (2008). The platelet-lymphocyte ratio improves the predictive value of serum CA19-9 levels in determining patient selection for staging laparoscopy in suspected periampullary cancer. Surgery.

[3] Forrest LM, McMillan DC, McArdle CS, Angerson WJ, Dunlop DJ (2003). Evaluation of cumulative prognostic scores based on the systemic inflammatory response in patients with inoperable non-small-cell lung cancer. Br J Cancer..

[4] Onodera T, Goseki N, Kosaki G (1984). Prognostic nutritional index in gastrointestinal surgery of malnourished cancer patients. Nippon Geka Gakkai Zasshi.

[5] Buzby GP, Mullen JL, Matthews DC, Hobbs CL, Rosato EF (1980). Prognostic nutritional index in gastrointestinal surgery. Am J Surg.

[6] Jian-Hui C, Iskandar EA, Cai ShI, Chen CQ, Chen CQ, Wu H (2016). Significance of Onodera's prognostic nutritional index in patients with colorectal cancer: a large cohort study in a single Chinese institution. Tumour Biol.

[7] Migita K, Takayama T, Saeki K, Matsumoto S, Wakatsuki K, Enomoto K (2013). The prognostic nutritional index predicts long-term outcomes of gastric cancer patients independent of tumor stage. Ann Surg Oncol.

[8] Kubo N, Ohira M, Tamura T, Sakurai K, Toyokawa T, Tanaka H (2017). Prognostic significance of baseline nutritional index for patients with esophageal squamous cell carcinoma after radical esophagectomy. Esophagus.

[9] Watanabe J, Otani S, Sakamoto T, Arai Y, Hanaki T, Amisaki M (2016). Prognostic indicators based on inflammatory and nutritional factors after pancreaticoduodenectomy for pancreatic cancer. Surg Today.

[10] Kwon WA, Kim S, Kim SH, Joung JY, Seo HK, Lee KH (2017). Pretreatment prognostic nutritional index is an independent predictor of survival in patients with metastatic renal cell carcinoma treated with targeted therapy. Clin Genitourin Cancer.

[11] Broggi MS, Patil D, Baum Y, Nieh PT, Alemozaffar M, Pattaras JG (2016). Onodera's prognostic nutritional index as an independent prognostic factor in clear cell renal cell carcinoma. Urology.

[12] Mohri T, Mohri Y, Shigemori T, Takeuchi K, Itoh Y, Kato T (2016). Impact of prognostic nutritional index on long-term outcomes in patients with breast cancer. World J Surg Oncol.

[13] Pinato DJ, North BV, Sharma R (2012). A novel, externally validated inflammation-based prognostic algorithm in hepatocellular carcinoma: the prognostic nutritional index (PNI). Br J Cancer.

[14] Chan AW, Chan SL, Wong GL, Wong VW, Chong CC, Lai PB (2015). Prognostic nutritional index (PNI) predicts tumor recurrence of very early/early stage hepatocellular carcinoma after surgical resection. Ann Surg Oncol.

[15] Okamura Y, Ashida R, Ito T, Sugiura T, Mori K, Uesaka K (2015). Preoperative neutrophil to lymphocyte ratio and prognostic nutritional index predict overall survival after hepatectomy for hepatocellular carcinoma. World J Surg.

[16] Goh BK, Kam JH, Lee SY, Chan CY, Allen JC, Jeyaraj P (2016). Significance of neutrophil-to-lymphocyte ratio, platelet-to-lymphocyte ratio and prognostic nutrition index as preoperative predictors of early mortality after liver resection for huge (≥10 cm) hepatocellular carcinoma. J Surg Oncol.

[17] Ji F, Liang Y, Fu S, Chen D, Cai X, Li S (2017). Prognostic value of combined preoperative prognostic nutritional index and body mass index in HCC after hepatectomy. HPB (Oxford).

[18] Kinoshita A, Onoda H, Imai N, Iwaku A, Oishi M, Fushiya N (2012). Comparison of the prognostic value of inflammation-based prognostic scores in patients with hepatocellular carcinoma. Br J Cancer.

[19] Huang J, Xu L, Luo Y, He F, Zhang Y, Chen M (2014). The inflammation-based scores to predict prognosis of patients with hepatocellular carcinoma after hepatectomy. Med Oncol.

[20] Zhang X, Li C, Wen T, Peng W, Yan L, Yang J (2017). Postoperative prognostic nutritional index predicts survival of patients with hepatocellular carcinoma within milan criteria and hypersplenism. J Gastrointest Surg.

[21] Wang Z, Wang J, Wang P (2018). The prognostic value of prognostic nutritional index in hepatocellular carcinoma patients: a meta-analysis of observational studies. PLoS ONE.

[22] Johnson PJ, Berhane S, Kagebayashi C, Satomura S, Teng M, Reeves HL (2015). Assessment of liver function in patients with hepatocellular carcinoma: a new evidence-based approach-the ALBI grade. J Clin Oncol.

[23] Ohkura Y, Mizuno S, Kishiwada M, Hamada T, Usui M, Sakurai H (2014). Benefit of technetium-99m galactosyl human serum albumin scintigraphy instead of indocyanine green test in patients scheduled for hepatectomy. Hepatol Res.

[24] Okuda Y, Mizuno S, Shiraishi T, Murata Y, Tanemura A, Azumi Y (2014). Clinicopathological factors affecting survival and recurrence after initial hepatectomy in non-B non-C hepatocellular carcinoma patients with comparison to hepatitis B or C virus. Biomed Res Int..

[25] Ogłuszka M, Orzechowska M, Jędroszka D, Witas P, Bednarek AK (2019). Evaluate cutpoints: adaptable continuous data distribution system for determining survival in Kaplan–Meier estimator. Comput Methods Programs Biomed.

[26] Kwon AH, Ha-Kawa SK, Uetsuji S, Inoue T, Matsui Y, Kamiyama Y (1997). Preoperative determination of the surgical procedure for hepatectomy using technetium-99m-galactosyl human serum albumin (99mTc-GSA) liver scintigraphy. Hepatology.

[27] Okabe H, Beppu T, Hayashi H, Mima K, Nakagawa S, Kuroki H (2014). Rank classification based on the combination of indocyanine green retention rate at 15 min and (99m)Tc-DTPA-galactosyl human serum albumin scintigraphy predicts the safety of hepatic resection. Nucl Med Commun.

[28] Ma C, Kesarwala AH, Eggert T, Medina-Echeverz J, Kleiner DE, Jin P (2016). NAFLD causes selective CD4(+) T lymphocyte loss and promotes hepatocarcinogenesis. Nature.

[29] Gabrielson A, Wu Y, Wang H, Jiang J, Kallakury B, Gatalica Z (2016). Intratumoral CD3 and CD8 T-cell densities associated with relapse-free survival in HCC. Cancer Immunol Res.

[30] Harimoto N, Shirabe K, Yamashita Y, Ikegami T, Yoshizumi T, Soejima Y (2013). Sarcopenia as a predictor of prognosis in patients following hepatectomy for hepatocellular carcinoma. Br J Surg.

[31] Yabusaki N, Fujii T, Yamada S, Suzuki K, Sugimoto H, Kanda M (2016). Adverse impact of low skeletal muscle index on the prognosis of hepatocellular carcinoma after hepatic resection. Int J Surg.

[32] Hamaguchi Y, Kaido T, Okumura S, Fujimoto Y, Ogawa K, Mori A (2015). Preoperative intramuscular adipose tissue content is a novel prognostic predictor after hepatectomy for hepatocellular carcinoma. J Hepatobiliary Pancreat Sci.

[33] Shiozawa S, Usui T, Kuhara K, Tsuchiya A, Miyauchi T, Kono T (2016). Impact of branched-chain amino acid-enriched nutrient on liver cirrhosis with hepatocellular carcinoma undergoing transcatheter arterial chemoembolization in barcelona clinic liver cancer stage B: a prospective study. J Nippon Med Sch.

[34] Yao H, Bian X, Mao L, Zi X, Yan X, Qiu Y (2015). Preoperative enteral nutritional support in patients undergoing hepatectomy for hepatocellular carcinoma: a strengthening the reporting of observational studies in epidemiology article. Medicine (Baltimore).

[35] Okabayashi T, Nishimori I, Sugimoto T, Maeda H, Dabanaka K, Onishi S (2008). Effects of branched-chain amino acids-enriched nutrient support for patients undergoing liver resection for hepatocellular carcinoma. J Gastroenterol Hepatol.

